# Model Analytical Development for Physical, Chemical, and Biological Characterization of* Momordica charantia* Vegetable Drug

**DOI:** 10.1155/2016/7528297

**Published:** 2016-08-07

**Authors:** Deysiane Oliveira Brandão, Geovani Pereira Guimarães, Ravely Lucena Santos, Fernando José de Lima Ramos Júnior, Karla Monik Alves da Silva, Fabio Santos de Souza, Rui Oliveira Macêdo

**Affiliations:** ^1^Department of Pharmaceutical Sciences, Postgraduate Program in Pharmaceutical Sciences, Federal University of Pernambuco, University City, Artur de Sá Avenue, 50740-521 Recife, PE, Brazil; ^2^Unified Laboratories of Pharmaceutical Development and Assays, Department of Pharmaceutical Sciences, Federal University of Paraíba, Campus I, University City, 58059-970 João Pessoa, PB, Brazil

## Abstract

*Momordica charantia* is a species cultivated throughout the world and widely used in folk medicine, and its medicinal benefits are well documented, especially its pharmacological properties, including antimicrobial activities. Analytical methods have been used to aid in the characterization of compounds derived from plant drug extracts and their products. This paper developed a methodological model to evaluate the integrity of the vegetable drug* M. charantia* in different particle sizes, using different analytical methods.* M. charantia* was collected in the semiarid region of Paraíba, Brazil. The herbal medicine raw material derived from the leaves and fruits in different particle sizes was analyzed using thermoanalytical techniques as thermogravimetry (TG) and differential thermal analysis (DTA), pyrolysis coupled to gas chromatography/mass spectrometry (PYR-GC/MS), and nuclear magnetic resonance (^1^H NMR), in addition to the determination of antimicrobial activity. The different particle surface area among the samples was differentiated by the techniques. DTA and TG were used for assessing thermal and kinetic parameters and PYR-GC/MS was used for degradation products chromatographic identification through the pyrograms. The infusions obtained from the fruit and leaves of* Momordica charantia* presented antimicrobial activity.

## 1. Introduction

The knowledge of traditional medicine and the use of medicinal plants are of fundamental importance in the development of new drugs by pharmaceutical companies because the plants, aside from their pharmacological applications, are likely to have low toxicity and are easy to access and of low cost [1]. In association with these facts, it must be considered that drugs already on the market are developing resistance to pathogens, causing serious problems for the world's population. Therefore, it is necessary to search for new therapeutic alternatives to treat these infections and to derive these alternatives mainly from natural products [2].

In this context,* Momordica charantia* is a species popularly known as “bitter gourd,” “bitter melon,” or “melão de são caetano.” This species is cultivated throughout the world and widely used in folk medicine, and its medicinal benefits are well documented, especially its pharmacological properties, including antimicrobial activities [3–5]. The extracts obtained from* Momordica charantia* fruit contain many biologically active metabolites including chemical glycosides, saponins, alkaloids, fixed oils, triterpenes, proteins, and steroids [3, 6]. Since this is a great source of plant phytochemicals, pharmacological and toxicological compounds may be tested and raw materials used for the production of a new herbal medicine with antimicrobial activity.

The market for herbal products is growing worldwide, but this increase is not reflected in the quality of available herbal medicines because there are major obstacles to effecting quality control, proof of safety, and proof of efficacy of herbal medicines. This is due to the chemical complexity of derivatives of vegetable drugs [7, 8].

Therefore, control of the granulometry of the powders from vegetable drugs is an important factor in the manufacturing process, as the particle size distribution is significantly influential in various stages of production [9]. In addition, in RDC number 26 of 13 May 2010, ANVISA [10] mentioned that one should consider the degree of reduction of particles in the case of medicinal teas or herbal drugs used as a final product by the consumer. Therefore, analytical methods have been used to aid in the characterization of compounds derived from plant drug extracts and their products.

In this sense, thermoanalytical techniques such as thermogravimetry (TG) and differential thermal analysis (DTA) are used for different purposes in pharmaceutical technology and are of fundamental importance in the characterization and thermal behavior of drugs such as those obtained from plant [11–14]. Pyrolysis coupled with gas chromatography and mass spectrometry (Pyr-GC/MS) is useful for elucidation of decomposition of complex volumes of organic molecules, such as in the case of vegetable drugs [15, 16]. Nuclear magnetic resonance, ^1^H NMR, has been applied successfully in several studies' fingerprinting, in which fingerprints of complex mixtures spectra under standard conditions are evaluated, since most organic metabolites contain the 1H isotope and have high natural abundance (99.98%) [17].

This paper developed a methodological model to evaluate the integrity of the vegetable drug* Momordica charantia* in different particle sizes, using different analytical methods in order to ensure the quality of the pharmaceutical plant's active ingredient.

## 2. Methodology

### 2.1. Obtaining the Vegetable Drug

*Momordica charantia* (leaves and fruits) was collected in the semiarid region of Paraíba, Brazil, in the countryside in São José da Mata, latitude 7°13′ and longitude 35°55′, between December 2014 and January 2015, respecting the ideal time for collection. The voucher specimen was deposited in the Herbário Manuel de Arruda Câmara (ACAM), State University of Paraíba, under registration number EAN-257/ACAM, thus proving the authenticity of the plant.

### 2.2. Preparation of Powder

The leaves and fruit of the plants were dehydrated in an oven with circulating air temperature controlled at 40°C. The dried material was milled using a mill with four mobile knives and four fixed Wiley models, Marconi brand, coupled to a sieve of 10 mesh. The powdered plant drug was subjected to a system of sieves with different pore sizes: >297 *μ*m, ≤297 *μ*m, ≤149 *μ*m, and ≤74 *μ*m. The fruits sample was denoted as F1 (>297 *μ*m), F2 (≤297 *μ*m), F3 (≤149 *μ*m), and F4 (≤74 *μ*m) and the leaves sample was denoted as P1 (>297 *μ*m), P2 (≤297 *μ*m), P3 (≤149 *μ*m), and P4 (≤74 *μ*m).

### 2.3. Thermogravimetry (TG) Analysis

Dynamic thermogravimetric curves of the powders of plants in different particle sizes were obtained in a Thermobalance TGA-50H model, Shimadzu, at a heating rate of 10°C/min, ranging from 25°C to a temperature of 900°C. We used an atmosphere of nitrogen and synthetic air with a flow of 50 mL/min and 20 mL/min, respectively. Mass used was 5.00 ± 0.05 mg, and each sample was packaged in an alumina crucible. The calibration of the thermobalance was performed using calcium oxalate monohydrate. The graphs were analyzed using TASYS software WS 60, Shimadzu, to characterize the weight loss steps.

### 2.4. Determination of the Kinetics of Degradation

The kinetics of degradation of the drug obtained from plant fruits and leaves of* M. charantia* were obtained using thermogravimetric curves in synthetic air atmosphere (20 mL/min) under heating rates of 5, 10, 20, and 40°C/min. Based on the data obtained in the gravimetric curves, we applied the Ozawa model to determine the kinetic parameters, reaction order (*n*), frequency factor (*A*), and activation energy (*E*_*a*_). For the determination of these parameters, we used TASYS software WS 60, Shimadzu.

### 2.5. Differential Thermal Analysis

Using the differential thermal curves, powders of plants in different particle sizes were obtained in a differential thermal analyzer, DTA-50, Shimadzu, at a heating rate of 10°C min^−1^, varying from 25°C to a temperature of 900°C under an atmosphere of nitrogen, with flow of 50 mL/min. The samples were placed in an alumina crucible using mass of 5.00 ± 0.05 mg for each sample. The calibration of the equipment was performed using melting point and enthalpy of indium and zinc as standards. The differential thermal curves were analyzed using the program TASYS 60, Shimadzu.

### 2.6. Pyrolysis Coupled with GC/MS

Pyrolysis was carried out using a pyrolyzer (Shimadzu, Pyr-4A), directly coupled to gas chromatography system/mass spectroscopy (Shimadzu GCMS-QP5050A). Capillary column with stationary phase was used: phenyl dimethylpolysiloxane (5 : 95) (30 m length, 0.25 mm internal diameter, and 0.25 particle size). The interface temperature was 70°C and was increased by a rate of 10°C/min to 300°C and then held for 5 minutes. Helium was used as a carrier gas at a flow rate of 1.7 mL/min and a split ratio of 1 : 5. The mass spectrometer was configured to scan a mass range from *m*/*z* 50 to *m*/*z* 450. The mass spectra were obtained by electron impact at an energy of 70 eV.

A sample consisting of a minuscule portion of powders of vegetal drugs was placed in a platinum crucible and introduced into the pyrolyzer at temperatures of 250, 350, and 450°C, selected according to the steps for displaying thermodecomposition by thermogravimetric analysis of the samples. Chemical compounds produced in pyrolytic processes were identified using comparative analysis with the mass spectra library of Wiley, 6th Edition, for Class-500.

### 2.7. Proton NMR Spectroscopy

H^1^-dimensional spectra were obtained using a Varian 500 MHz spectrometer with a sample of waste evaporated hydroethanolic extract (HSS) of vegetable drugs in different particle sizes, solubilized in deuterated DMSO (DMSO-d6). We then compared the intensities of the analytical signal in different particle sizes under the same extraction conditions.

Then, 5 mL of 50% hydroethanol solution was added. The assembly was subjected to extraction in an ultrasound bath (USC 2800A, Unique, Brazil) for 1 hour and then left to stand for 24 hours. The extractive cycle was repeated three times. Then, the material was filtered using cotton. The filtrate was placed in a porcelain capsule, and the solvent was evaporated in an oven with air circulation (ET 394/2, Tecnal, Brazil) at 40°C for 24 hours. At the end of the process, we were left with a residue which was solubilized in DMSO-d6 and subjected to analysis.

All spectra were manually corrected for phase and baseline and analyzed using the MestreNova® 6.0.2 software (Mestrelab Research). Chemical shifts (*δ*) are expressed in parts per million (ppm).

### 2.8. Determination of the Antimicrobial Activity

To evaluate the antimicrobial activity, 5% infusions were obtained from different particle sizes of the fruits and leaves. Standard strains used included American Type Culture Collection (ATCC) of* Escherichia coli* (ATCC 25922),* Staphylococcus aureus* (ATCC 25923),* Pseudomonas aeruginosa* (ATCC 27853), and* Klebsiella pneumoniae* (ATCC 4352), which were made available by the Oswaldo Cruz Foundation (Fiocruz, RJ).

#### 2.8.1. Screening Microbiology

The microorganisms were maintained in test tubes containing BHI agar and then grown in Holiday Rentals Abroad Hinton agar at 37°C for 24 hours. The determination of minimum inhibitory concentration (MIC) was performed by broth microdilution method. It was performed in 96-well microplates according to the Clinical and Laboratory Standards Institute (CLSI) procedures [18].

The inocula were standardized into tubes containing 5 mL sterile 0.9% saline. The suspension was adjusted by spectrophotometer at 625 nm in a concentration equivalent to 10^6^ UFC.

One hundred microliters of infusion of 5% (50 mg/mL) was subjected to serial dilution in Müller-Hinton broth in each microplate, thereby obtaining the following concentrations: 25, 12.5, 6.25, 3.12, 1.56, 0.78, 0.39, and 0.195 mg/mL in each infusion. Ten microliters of each microculture was added to each well of the microplate.

They were used as negative control, and distilled water was used as the positive control, gentamicin 80 mg/mL. The bacterial growth was indicated by addition of 20 *μ*L of aqueous resazurin (Sigma-Aldrich) 0.01%, with further incubation at 37°C ± 1°C for 2 h. Viable microorganisms reduced the blue dye to a pink color. Thus, the MIC was defined as the lowest concentration at which there was no change of color noted in the dye.

## 3. Results and Discussion

### 3.1. Thermogravimetric Analysis (TGA)

**Figure 1:**
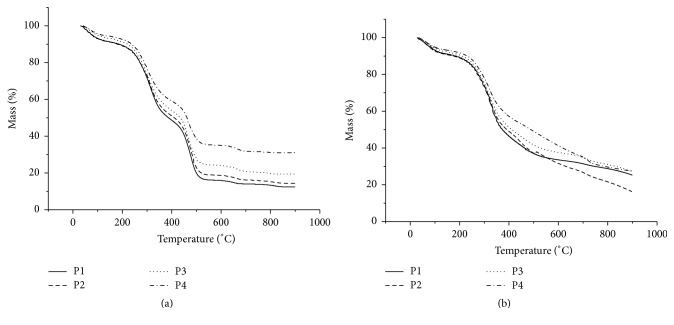
Drug* Momordica charantia* (leaves) TG curves in different particle sizes at a heating rate of 10°C min^−1^ in synthetic air (a) and inert atmosphere (b).

**Figure 2:**
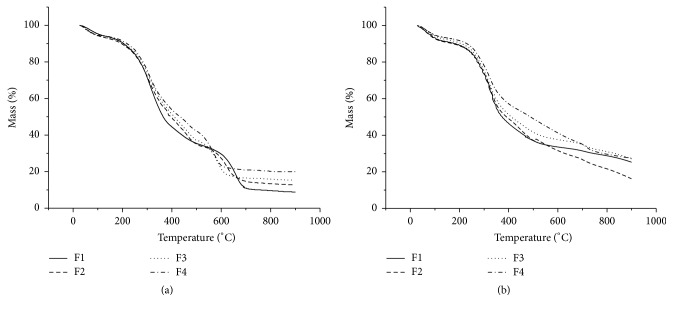
Drug* Momordica charantia* (fruits) TG curves in different particle sizes at a heating rate of 10°C min^−1^ in synthetic air (a) and inert atmosphere (b).

Thermal decomposition of the samples, both leaves and fruit, occurred in six stages when subjected to the synthetic air atmosphere at a heating ratio of 10°C/min. However, it was observed that the presence of synthetic air degradation of the vegetable drugs occurred more intensely, thus presenting a further step of degradation and a minor mineral residue in all the samples as compared to inert atmosphere at the same rate of heating (Figures 1 and 2).

In an oxidative atmosphere (synthetic air), thermal decomposition of the first event occurred at the following temperatures ranges in the leaves: 35–103°C, 35–106°C, 35–109°C, and 35–116°C, with the following losses of mass: 6.7%, 7.3%, 5.6%, and 5.0% for P1, P2, P3, and P4 samples, respectively. The same event was observed when plants were subjected to an inert atmosphere, thereby obtaining the following temperature ranges: 35–96°C, 35–105°C, 35–106°C, and 35–107°C, for the following losses of equivalent mass: 3.3%, 6.9%, 6.1%, and 5.4%, referring to samples P1, P2, P3, and P4, respectively. For vegetable drugs obtained from the fruits of* Momordica charantia,* we observed similar events. Thus, this first stage of decomposition occurred in synthetic air at 35–108°C, 35–90°C, 35–89°C, and 35–116°C with the mass losses of 4.6% for samples F1 and F2 and 4.7% to 5.0% for F3 and F4, respectively, according to the same events as vegetable drugs were subjected to an inert atmosphere, thereby obtaining the following temperature ranges: 35–107°C, 35–108°C, 35–110°C, and 35–114°C for mass losses equal to 3.8%, 4.5%, 5.3%, and 4.4%, referring to samples F1 F2, F3, and F4, respectively.

The first step of thermal decomposition is to hallmark the loss of volatile products, mainly free water, as this event occurs in fusion tracks and with vaporization of the substance [19, 20]. These weight loss values are related to water, and the recommended level of water in the sample is less than 14% [21].

The second stage of thermal degradation is related to the loss of bound water in vegetable drugs. An increased mass loss was observed in vegetable drugs obtained from fruits (F1, F2, F3, and F4) with losses of 2.2%, 3.2%, 3.1%, and 4.0%, respectively, with respect to the obtained sheets (P1, P2, P3, and P4) with loss of 4.5%, 4.4%, 4.6%, and 4.0%, respectively. Almost the same events were observed in the two atmospheres evaluated, indicating that perhaps this degradation step does not depend on the atmosphere to which the vegetables drugs were subjected.

In plant drugs obtained from* Momordica charantia* leaves, the third stage in synthetic air introduced the maximum value of mass loss of 11.9%, occurring between 213°C and 285°C for sample P1, obtaining similar values in an inert atmosphere. For drugs derived from the fruit, the greatest mass loss in oxidative atmosphere occurred in sample F3 between the temperature ranges of 184°C to 267°C, with 9.3% mass loss occurring when subjected to similar values in an inert atmosphere. It is suggested that in the decomposition there was no degradation of matrix components of the plant, only output samples of the constituents; there may have been a phase transition, such as volatilization, since similar profiles were obtained for vegetable drugs studied in two atmospheres [22].

The stage of degradation in which there was the greatest weight loss was the fourth step for all herbal drugs in the study. Thus, among the drugs of the obtained sheets that had more significant degradation was P2 with a weight loss of 24.6% obtained between temperatures of 270°C to 358°C. For the fruit that had higher degradation at this stage, F1 was the highest, with mass loss of 35.9% between temperature ranges of 251°C to 360°C. This stage was regarded as the primary stage of thermal decomposition of all plant drugs. This event is likely associated with the thermal decomposition of carbohydrates and other organic compounds present in the species [23].

The thermal decomposition in the two profiles in similar atmospheres was evaluated in this step, as were samples obtained from the fruit and leaves and different particle sizes, thus suggesting the presence of the same chemical constituents of organic origin in all plant drugs.

The following steps represent the continuation of the degradation process with mineral residue formation in the end. The quantity of the mineral residue ranged from 18.3% to 37.0% for the samples obtained from the leaves and 10.2% to 37.0% for the samples obtained from the fruit. Also observed was variation according to particle size. The data demonstrate that mineral waste samples of leaves and fruits in different particle sizes can be used to indicate the integrity of the sample for each particle size differentiation. These results corroborate those found by Correia et al., 2016 [20].

### 3.2. Kinetic Parameters Determination

The fourth step was chosen as that in which the major weight loss in the thermal decomposition reaction process for all samples occurred and was used to calculate the kinetic parameters of the degradation using the Ozawa method. Data for this step are described in Tables 1 and 2.

Thus, thermogravimetric curves of all the samples were obtained following the heating rates 40, 20, 10, and 5°C/min. All curves were obtained using the oxidative atmosphere.

**Figure 3:**
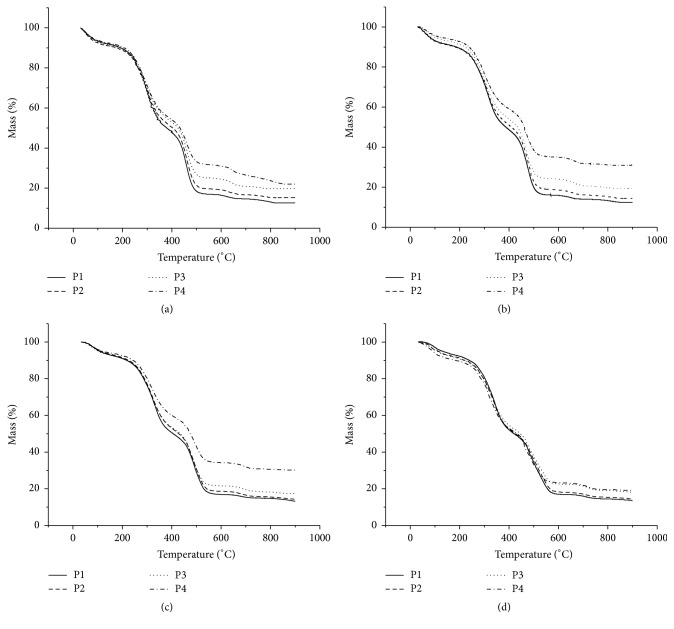
*M. charantia* (leaves) TG curves in different particle sizes. (a) Rate of 5°C/min. (b) Rate of 10°C/min. (c) Rate of 20°C/min. (d) Rate of 40°C/min.

**Figure 4:**
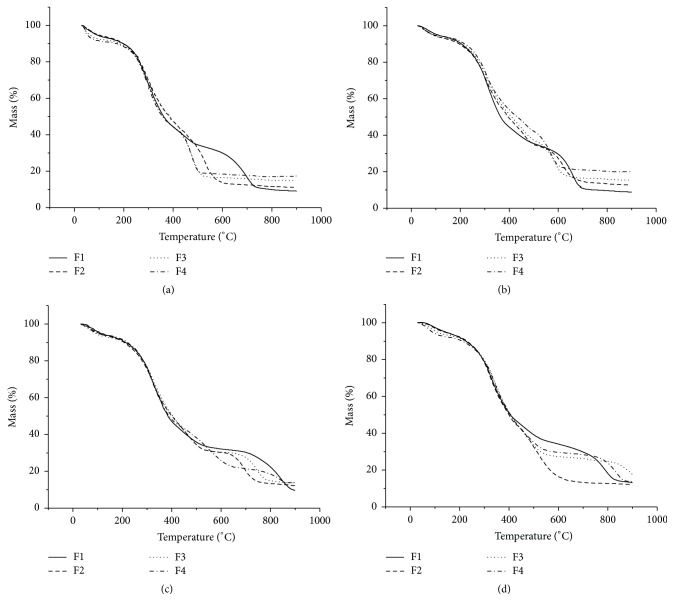
*M. charantia* (fruits) TG curves in different particle sizes. (a) Rate of 5°C/min. (b) Rate of 10°C/min. (c) Rate of 20°C/min. (d) Rate of 40°C/min.

All samples demonstrated six thermal decomposition events. Increasing initial temperature of each event as a function of heating rate was observed. There was also displacement of the temperatures and mass loss according to particle size when heating for the same reason (Figures 3 and 4). The particles of smaller size (P4 and F4) had a smaller loss of mass, thus resulting in greater mineral residue.

This displacement may be associated with different chemical composition in each particle size. There are different heat transfer rates for each sample and there is different activation energy for degradation at each different size of particle [24, 25]. Different profiles of the drugs were obtained from the* M. charantia* leaves and fruit.

The greatest weight loss was observed in sample P1 when subjected to heating rate of 5°C/min, and this mass loss was equal to 29.4%, occurring between the temperatures of 247.65°C to 342.13°C. This greater weight loss can be associated with the presence in sample P1 of a greater amount of chemical constituents degradable at this temperature range. This was similar to the F1 sample at a rate of 10°C/min, which was the sample between herbal drugs obtained from fruits that demonstrated greater weight loss (35.974%) between 251.66°C and 360.03°C.

With the calculation obtained using the kinetic method of Osawa, the same order of reaction for all samples was given, as shown in Table 3.

It was observed that the value of the activation energy decreases with decreasing particle size. This occurred both in the fruit samples obtained and in samples obtained from the leaves. Thus, it was observed that the activation energy followed the order F1 > F2 > F3 > F4 in the fruits and P1 > P2 > P3 > P4 in the leaves. The activation energy is defined as the minimum energy required to cause chemical reactions, including cracking reactions of macromolecules [26]. Thus, this result was expected since the powders of samples with smaller particle size were characterized by having a larger surface area and were more unstable samples, therefore requiring lower activation energy for the reactions of thermodecomposition to occur [27].

### 3.3. Differential Thermal Analysis

The vegetable drugs obtained from the leaves of* M*.* charantia* when subjected to differential thermal analysis showed two exothermic events (Tables 4 and 5).

It was observed that the second event had higher energy release, thus suggesting that this event is related to a more significant weight loss. This suggestion is supported by correlating the data with the DTA curve of the TG curves, noting that this event with greater energy release occurred immediately after the greatest mass loss in the thermogravimetric analysis step. In this temperature range where there is degradation of aromatic and saturated carbon atom-linked structures, other breaches of hydrocarbon structures are present in large quantities in vegetable drugs [23].

Moreover, it was observed that in the second exothermic peak there was a proportional decrease in enthalpy as a function of the particle sizes; thus, the obtained sample P4 demonstrated lower enthalpy. This difference in energy values may be associated with the presence of macrocomponents such as cellulose and lignins which may occur in larger quantities in samples with larger particle sizes, thus requiring greater energy release in order to degrade these components [28].

For drug obtained fruit was observed of similar profiles (Table 5) in the DTA curves, thus presenting two exothermic peaks, the second event with greater enthalpy, correlating with the thermogravimetric curves where there is a greater mass loss.

The fruit vegetable drugs showed a higher enthalpy variation in the second event in accordance with the particle size; thus, F4 sample with the smallest particle size demonstrated lower enthalpy in this second peak.

### 3.4. Pyr-GC/MS

The data of TG curves demonstrates significant mass losses of vegetable drugs in the temperature range 250°C to 500°C. In order to assess which degradation products were being released, the samples were subjected to the pyrolysis process at temperatures of 250°C, 350°C, and 450°C, representing the intermediate and initial temperatures of the thermal decomposition process. In Tables 6 and 7, the main compounds released during the stages of thermal degradation can be seen, analyzed using the library of Wiley, 6th Edition, for Class-500.

**Figure 5:**
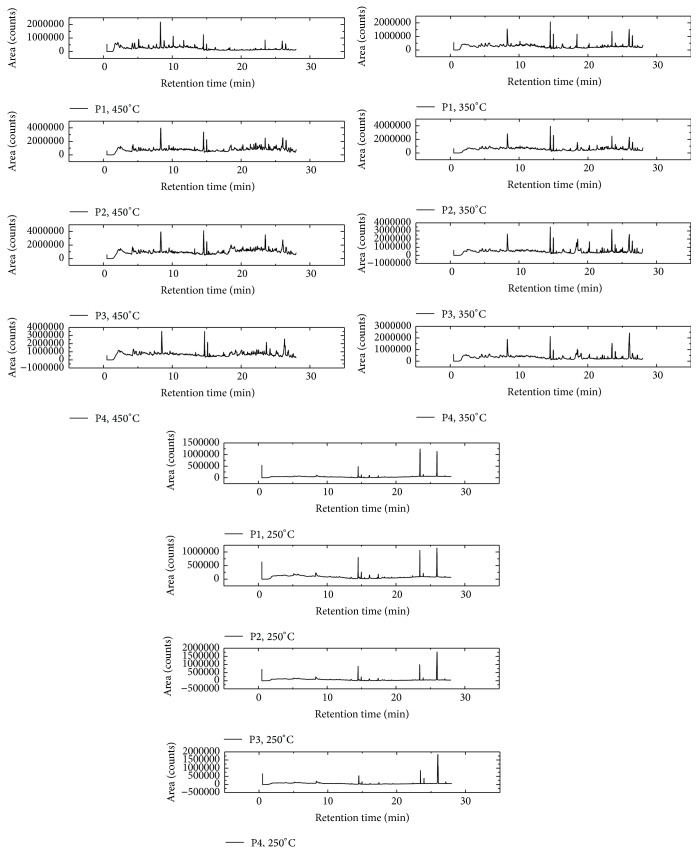
*Momordica charantia* leaves pyrograms obtained in different particles sizes and at the temperatures of 250, 350, and 450.

**Figure 6:**
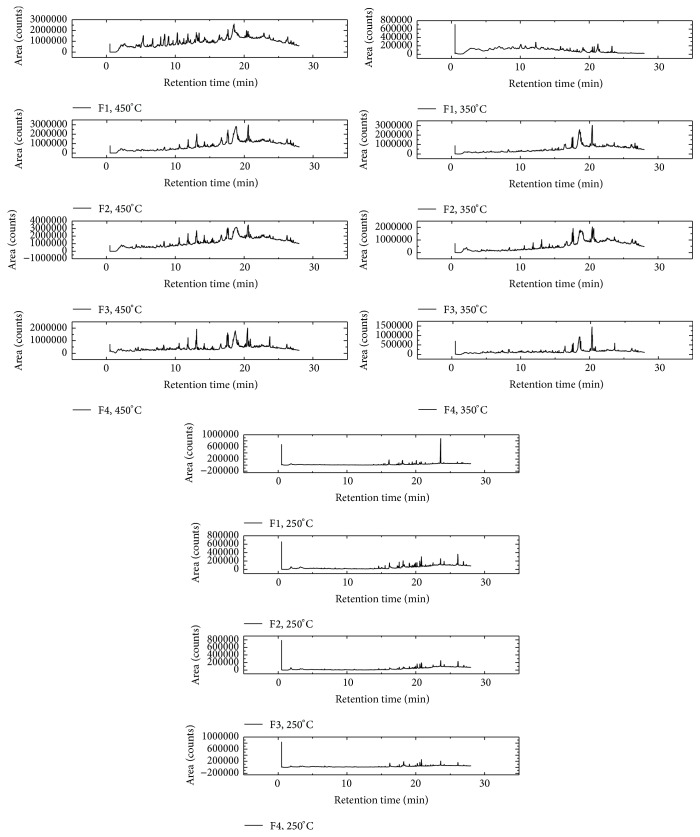
*Momordica charantia* fruits pyrograms obtained in different particles sizes and at the temperatures of 250, 350, and 450.

With the data in Tables 6 and 7, one realizes that the same components, in certain temperatures, were released from all fruit particle sizes and did not just occur in differentiation between drugs of different grain size in relation to the compounds analyzed. There were different intensity values and chromatographic peaks that varied according to particle size. The same thing happened with the herbal drugs obtained from the leaves (Figures 5 and 6).

Samples of leaves from the neophytadiene substance (C_20_H_38_) were evident at all three temperatures, but with different areas, with higher values of area under the temperature of 450°C, and with 16.3 min retention time for retaining P1, 25.8 min for P2, 26.8 min for P3, and 27.2 min for P4. Other compounds also appeared to be highlighted in these samples, such as solanesol (C_45_H_74_O), which was observed at temperatures of 350°C and 450°C, and myristic acid (C_14_H_28_O_2_) and limonene (C_10_H_16_) evident at temperatures of 350°C and 450°C, respectively. Samples obtained from fruits submitted to 250°C had similar compounds that showed larger areas, including stearic acid (C_18_H_36_O_2_) and palmitic acid (C_16_H_32_O_2_), highlighting retention times of 24.7 min for F1, 25.3 min for F2, 25.4 min for F3, and 25.8 min for F4, and showed palmitic acid for F1 at 26.0 min, 26.5 min for F2 and F3, and 26.6 min to 26.4 min for F4.

The stearic acid was also evident at a temperature of 350°C, but with a greater area at 22.3 min retention time for F1, 22.4 min for F2, 22.8 min for F3, and 22.9 min for F4. Besides this substance, myristic acid also achieved considerable intensity, appearing at 18.5 min retention time, 18.6 min, and 18.7 min, and 18.1 min for samples F1, F2, F3, and F4, respectively. At a temperature of 450°C, the substances with higher intensity were oleic acid, limonene, and eugenol, with greater intensity demonstrated in F1 retention time of 17.3 min, 10.1 min, and 14.1 min, respectively.

For purposes of quantitative analysis of the released compounds, the relative area of each substance in different particle size at each temperature was calculated. To calculate the relative area of similar peaks for the three samples, the sum of the areas of all the compounds analyzed was considered as 100%.

For the samples obtained from the leaves was verified through these percentages than sample whose particle size is smaller (P4) showed a area chromatographic smaller considering the compound neophytadiene (C_20_H_38_) when related to particle sizes larger, noted this profile at the three temperatures analyzed. Various profiles of relative area were observed in other compounds.

With the obtained samples of the fruits, it was also observed that the sample with the smallest particle size (F4) was obtained on a smaller area peak when considering some compounds. At temperatures of 250°C to 350°C, this profile prevailed for compounds stearic acid and palmitic acid. In the 450°C temperature profile, it was observed that, in the compounds of palmitic acid and oleic acid, various profiles of relative area were observed in other compounds.

It was also observed that, at a temperature of 450°C, chromatographic peaks resulting from the pyrolytic process showed the presence of a large number of substances from the thermal degradation. At temperatures slightly less than this, a greater loss of mass thermal analysis (TG) and increase in the release of energy in differential thermal analysis (DTA) are expected. Thus, the degradation products are detected in larger amounts at this temperature.

Therefore, the results showed that the samples of* M. charantia* in different particle sizes can be differentiated by degradation pyrograms obtained at temperatures of 250, 350, and 450°C.

### 3.5. Proton NMR Spectroscopy

The characterization and structural elucidation, chemical modification, biological activity, and the study of bioactive metabolites of mechanism of action were always made after the purification of the metabolites. However, the processes for obtaining these compounds in pure form are very long and tedious. Thus, it is of great value to develop methodologies that are able to relate the information about the chemical composition of a crude extract [29].

Thus, the ^1^H NMR analysis was used in an attempt to highlight possible similarities and differences among the samples with respect to particle size. Thus, compared to the quantitative intensity of peaks in different particle sizes, in Table 8, we can see the number of peaks per chemical shift of the spectra of each sample.

The numbers of the peaks vary with each plant sample, showing that samples with smaller particle sizes provide more peaks. The variation in the amount of surge can be justified by the presence and quantity of chemical compounds that can vary between samples, whereas the samples with smaller particle sizes can appear to have a greater amount of soluble substance in the used solvents (EtOH and deuterated DMSO) [30].

The peaks evident in the area of 0.0–3.0 can be attributed to the presence of aliphatic, methyl, and methylene groups, carboxylic groups, and aminic and amidic groups, while the region of 3.0–6.0 can be attributed to amino acid and compounds in the glycosylated region from 6.0 to 9.0, with aromatic and phenolic compounds [30].

It was also observed that the region where there were a larger number of peaks was the region 0.0–3.0 ppm. Some studies claim that various chemical compounds can be identified in this region, such as triterpene glycosides and steroids [30–32]. Studies are reported in the literature using the same technique and other more sensitive techniques which precisely identify such structures in* Momordica charantia*, both on leaves and on fruits [33–38]. There were also a greater number of drug peaks in this region obtained from fruits than in drugs obtained from leaves; there may therefore be a greater number of such structures in the* Momordica charantia* fruit

### 3.6. Determination of Antimicrobial Activity

In the microbiological screening of* M. charantia*, the plant drug was found to show antimicrobial activity against standard strains of* S. aureus*,* E. coli*,* P. aeruginosa*, and* K. pneumoniae*, demonstrating that water is an effective solvent which can be used for the extraction of bioactive plant materials (Table 9). This information is important because, according to some studies [39], water is usually the primary solvent used in folk medicine to obtain preparations from plants. Many of these preparations are used to treat conditions which would normally result in bacterial infections.

In Table 9, the values of MIC of materials infused at concentrations below 1 mg/mL for different bacteria, regardless of particle sizes, can be seen. However, there are differences in MIC values for the infused leaves among bacteria, with the more potent ones in* Staphylococcus aureus* and* Escherichia coli* and the less potent ones in* Pseudomonas aeruginosa* and* Klebsiella pneumoniae*. The infused fruits showed MIC values with more power for* Escherichia coli*, followed by* Klebsiella pneumoniae,* and less potency in* Staphylococcus aureus* and* Pseudomonas aeruginosa*. Particle sizes influenced only the microbial potency of* Escherichia coli*. Studies report varied chemical composition and pharmacological activity as a function of particle size [22, 28].

## 4. Conclusions

The characterization of vegetables drugs obtained from* Momordica charantia* has been specified according to the size of particles through the thermal and kinetic data obtained from TG and DTA. Chromatographic data, in relation to the peak areas of the examined compounds, showed quantitative differences in the chemical composition of the fruit and leaves of* Momordica charantia*, as well as in different particle sizes.

These results were confirmed by data obtained by proton NMR showing peak quantities that varied according to the plant sample. Demonstrating the potential of proton NMR spectroscopy as an analytical tool with its characteristics, this is nonspecific, inexpensive, and not requiring long time for preparing the sample. This technique may thus have new applications and be a tool for characterization of raw samples.

The infusions obtained from the fruit and leaves of* Momordica charantia* presented antimicrobial activity against* S. aureus*,* P. aeruginosa*,* E. coli*, and* K. pneumoniae*, obtaining lower MIC values than 1 mg/mL in all particle sizes.

The methodological model developed was satisfactory to differentiate vegetables drugs in different particle sizes of leaves and fruits of* Momordica charantia.*

## Figures and Tables

**Table 1:** Decomposition of the main parameters of the samples obtained from the degradation step leaves.

Samples	Rate (°C/min)	Onset (°C)	End (°C)	Weight loss (%)
P1	5	247,65	342,13	29,4
	10	285,06	347,95	20,95
	20	286,29	364,07	21,099
	40	291,74	374,78	23,68

P2	5	246,79	345,33	27,622
	10	270,67	358,18	24,601
	20	285,68	364,43	23,33
	40	228,11	377,00	25,461

P3	5	258,8	341,54	23,086
	10	267,41	352,54	23,145
	20	279,68	373,59	25,48
	40	315,01	387.98	19,111

P4	5	262,36	350,7	22,35
	10	264,9	355,38	21,994
	20	292,08	373,47	19,082
	40	298,77	376,04	21,825

**Table 2:** Decomposition of the main parameters of the samples obtained from the degradation step fruits.

Samples	Rate (°C/min)	Onset (°C)	End (°C)	Weight loss (%)
F1	5	248,19	356,07	32,764
	10	251,66	360,03	35,974
	20	263,18	364,96	29,223
	40	295,15	393,36	28,788

F2	5	215,86	341,84	26,699
	10	270,67	344,99	24,985
	20	251,16	362,28	27,477
	40	275,13	369,21	27,185

F3	5	238,7	351,89	32,449
	10	267,22	380,08	28,589
	20	401,51	586,96	18,052
	40	407,77	587.98	19,111

F4	5	259,77	361,88	30,83
	10	343,61	393,11	24,842
	20	362,73	425,92	10,933
	40	401,51	527,7	34,756

**Table 3:** Thermal decomposition kinetic parameters (fruits and leaves).

	Kinetic parameters		
	Activation energy (kJ/mol)	Reaction order	Frequency factor (min^−1^)
F1	137,23	0	2,409 × 10^7^
F2	132,35	0	1,376 × 10^6^
F3	79,54	0	4,227 × 10^3^
F4	64,71	0	6,112 × 10^2^
P1	217,38	0	1,648 × 10^14^
P2	180,81	0	2,867 × 10^11^
P3	168,12	0	3,426 × 10^10^
P4	88,71	0	3.265 × 10^6^

**Table 4:** Exothermic events of DTA (leaves).

Samples	1° event		2° event	
	Temperature of peak (°C)	Enthalpy (kJ/g)	Temperature of peak (°C)	Enthalpy (kJ/g)
P1	315,86	0,464	456,90	4,18
P2	316,28	0,433	457,74	3,78
P3	328,21	0,445	457,26	3,42
P4	333,57	0,416	458,51	2,71

**Table 5:** Exothermic events of DTA (fruits).

Samples	1° event		2° event	
	Temperature of peak (°C)	Enthalpy (kJ/g)	Temperature (°C)	Enthalpy (kJ/g)
F1	350,65	0,526	461,98	2,33
F2	353,79	0,521	462,34	2,24
F3	339,75	0,509	457,26	2,12
F4	336,96	0,505	458,51	1,46

**Table 6:** Identified substances obtained in different temperatures (*M. charantia* leaves).

Temperature	Substance	Samples											
		P1			P2			P3			P4		
		Area	%	Rt. (min)	Area	%	Rt. (min)	Area	%	Rt. (min)	Area	%	Rt. (min)
250°C	Neophytadiene (C_20_H_38_)	2850,342	45,87	25,8	2692,730	44,90	26,8	563,273	4,32	27,2	2383,374	43,08	27,4
	Palmitic acid (C_16_H_32_O_2_)	1583,093	25,47	23,2	1559,289	26,00	23,5	1539,281	26,58	24,6	1510,295	24,70	24,9
	n-Hexatriacontane (C_36_H_74_)	993,024	15,98	12,8	974,583	16,25	12,9	948,215	16,37	13,4	918,271	16,60	13,6
	Farnesol (C_15_H_26_O)	787,294	12,67	14,9	769,247	12,82	15,1	738,458	12,75	15,2	719,342	13,00	15,5

350°C	Neophytadiene (C_20_H_38_)	2903,483	24,38	26,1	2748,301	23,66	26,4	2573,293	22,78	26,7	2357,492	21,65	27,1
	Palmitic acid (C_16_H_32_O_2_)	1780,329	14,95	23,4	1754,382	15,10	23,8	1738,962	15,39	24,2	1712,227	15,72	24,6
	n-Hexatriacontane (C_36_H_74_)	1291,548	10,84	12,7	1267,265	10,91	13,1	1243,837	11,01	13,4	1205,381	11,07	13,8
	Farnesol (C_15_H_26_O)	782,391	6,57	15,1	763,274	6,57	15,4	740,394	6,55	15,7	714,392	6,56	15,9
	Solanesol (C_45_H_74_O)	1882,761	15,81	8,3	1862,397	16,03	8,5	1846,286	16,34	9,1	1829,265	16,80	9,3
	Myristic acid (C_14_H_28_O_2_)	1090,284	9,15	17,8	1074,374	9,25	18,2	1047,213	9,27	18,3	1018,537	9,35	18,7
	Pyrogallol (C_6_H_3_(OH)_3_)	1284,325	10,78	19,8	1265,539	10,89	20,1	1248,297	11,05	20,5	1222,389	11,22	20,8
	Oleic acid (C_18_H_34_O_2_)	893,258	7,50	16,1	876,290	7,54	16,3	854,261	7,56	17,1	827,294	7,59	17,2

450°C	Neophytadiene (C_20_H_38_)	2953,257	20,35	26,1	2725,283	19,49	26,5	2498,273	18,73	26,8	2229,038	17,60	27,3
	Oleic acid (C_18_H_34_O_2_)	783,221	5,39	16,5	753,938	5,39	16,7	721,883	5,41	17,1	709,216	5,60	17,6
	Chloro-octadecanol (C_18_H_37_Cl)	1190,382	8,20	22,1	1174,398	8,40	22,4	1144,829	8,58	22,6	1119,503	8,84	23,1
	Carvacrol (C_10_H_14_O)	984,120	6,53	3,6	963,825	6,89	3,8	932,168	6,99	4,2	911,274	7,19	4,6
	Palmitic acid (C_16_H_32_O_2_)	1589,251	10,95	23,5	1565,390	11,20	23,8	1534,027	11,50	24,1	1515,356	11,96	24,4
	Farnesol (C_15_H_26_O)	692,383	4,77	14,7	674,372	4,82	15,1	655,219	4,91	15,5	627,391	4,95	15,8
	Myristic acid (C_14_H_28_O_2_)	794,276	5,47	17,7	771,264	5,51	18,2	742,111	5,56	18,6	718,432	5,67	19,1
	Solanesol (C_45_H_74_O)	2576,324	17,76	15,6	2465,219	17,63	16,1	2298,346	17,23	16,5	2093,287	16,53	16,8
	Pyrogallol (C_6_H_6_O_3_)	1398,281	9,36	19,5	1372,901	9,82	19,7	1343,205	10,07	20,2	1311,361	10,35	20,1
	Limonene (C_10_H_16_)	882,298	6,08	13,2	861,295	6,16	13,7	836,273	6,27	13,9	819,375	6,47	14,1
	m-Cresol (C_7_H_8_O)	662,348	4,56	5,1	648,271	4,63	5,3	629,112	4,71	5,7	608,351	4,80	6,1

Rt.: retention time.

**Table 7:** Identified substances obtained in different temperatures (*M. charantia* fruits).

Temperature	Substance	Samples											
		F1			F2			F3			F4		
		Area	%	Rt. (min)	Area	%	Rt. (min)	Area	%	Rt. (min)	Area	%	Rt. (min)
250°C	Stearic acid (C_18_H_36_O_2_)	1637,234	50,8	24,7	976,990	48,70	25,3	965,899	48,51	25,4	879,422	48,27	25,8
	Propionic acid (C_18_H_26_O_3_)	508,234	21,7	16,2	501,543	24,78	16,5	490,700	24,74	16,6	424,876	23,44	16,9
	Palmitic acid (C_16_H_32_O_2_)	346,521	16,9	26,0	323,987	16,00	26,5	310,887	15,67	26,6	299,563	15,22	26,4

350°C	3-Methyl-cyclohexane (C_7_H_14_O)	233,321	10,6	16,1	221,344	10,93	16,4	215,543	10,86	16,7	208,742	21,65	16,9
	Palmitic acid (C_16_H_32_O_2_)	1011,543	22,30	23,4	1000,257	21,45	23,8	987,213	21,37	24,2	939,458	15,72	23,9
	Phytol (C_40_H_20_O)	689,456	14,26	18,7	650,854	13,96	18,1	634,739	14,94	18,4	617,947	11,07	18,6
	Stearic acid (C_18_H_36_O_2_)	1876,675	38,80	22,4	1776,946	38,12	22,8	1564,987	35,60	22,9	1482,432	6,56	22,9
	Myristic acid (C_14_H_28_O_2_)	1832,456	18,5	18,3	1817,587	18,6	8,5	1806,637	18,85	18,7	1794,322	16,80	19,1
	n-Eicosanol (C_20_H_42_O)	423,876	8,76	13,8	375,686	8,91	14,2	401,543	9,24	14,3	376,836	9,35	14,9

450°C	Palmitic acid (C_16_H_32_O_2_)	1587,529	15,72	23,1	1573,478	15,56	23,5	1546,851	15,07	23,8	1515,876	14,62	24,2
	Cyclohexanol-3-methyl (C_7_H_14_O)	876,934	8,07	16,5	852,129	8,16	16,7	833,990	8,39	17,1	809,231	8,39	17,3
	Guaiacol (C_2_H_2_O_2_)	598,976	5,51	8,1	567,306	5,43	8,4	529,408	5,32	8,6	506,096	5,24	8,9
	Myristic acid (C_14_H_28_O_2_)	1798,903	16,57	18,5	1764,807	16,90	18,8	1749,091	17,59	4,2	1700,093	17,63	19,6
	n-Eicosanol (C_20_H_42_O)	398,446	3,67	13,5	371,913	3,56	13,8	346,021	3,48	14,1	332,765	3,45	14,4
	Eugenol (C_10_H_12_O_2_)	786,034	7,24	11,7	772,865	7,40	12,1	743,294	7,47	12,5	722,341	7,49	12,7
	Oleic acid (C_18_H_34_O_2_)	2657,897	24,48	22,7	2447,381	23,44	22,2	2166,823	21,80	22,6	2096,379	21,74	22,7
	Methyl pulegenato (C_6_H_14_O)	689,028	6,34	15,4	653,987	6,26	16,1	626,731	6,30	16,2	608,355	6,31	16,3
	Limonene (C_10_H_16_)	973,254	8,96	13,7	964,211	9,23	14,2	943,598	9,49	14,6	921,111	9,55	14,5
	Solanesol (C_45_H_74_O)	487,675	4,49	10,2	471,991	4,52	10,7	452,343	4,55	10,9	427,965	4,43	10,2

Rt.: retention time.

**Table 8:** More evident peaks in the ^1^H NMR spectrum of *Momordica charantia*.

Samples	Number of peaks by region				Total
	0,0–3,0 (ppm)	3,0–6,0 (ppm)	6,0–9,0 (ppm)	9,0–14,0 (ppm)	
F1	46	69	35	15	165
F2	47	71	41	18	177
F3	51	73	52	21	197
F4	58	78	55	22	213
P1	51	57	40	19	167
P2	59	64	49	21	193
P3	62	67	42	24	195
P5	66	71	41	25	203

**Table 9:** Antimicrobial activity of plants tested against microorganisms.

Herbal drugs	MIC (mg/mL) Microorganisms tested			
*Momordica charantia *leaves	*S.a.*	*E.c.*	*P.a.*	*K.p.*
P1	0,195	0,195	0,780	0,780
P2	0,195	0,195	0,780	0,780
P3	0,195	0,195	0,780	0,780
P4	0,195	0,390	0,780	0,780
*Momordica charantia *fruits				
F1	0,780	0,195	0,780	0,390
F2	0,780	0,390	0,780	0,390
F3	0,780	0,390	0,780	0,390
F4	0,780	0,195	0,780	0,390

*S.a.: Staphylococcus aureus; E.c.: Escherichia coli; P.a.: Pseudomonas aeruginosa; K.p.: Klebsiella pneumonia*.
